# Association and discriminative value of insulin resistance surrogate markers for aortic stiffness in asymptomatic adults

**DOI:** 10.3389/fcvm.2026.1920101

**Published:** 2026-09-28

**Authors:** Zhensen Zhou, Yang Wan, Yin Cheng, Yushan Tang, Xiaoyu Zhang, Zuchang Ma, Wanqiu Zhang, Liuhui Zhang

**Affiliations:** 1 Adult Infusion Department, The First Affiliated Hospital of Anhui Medical University, Hefei, China; 2 Department of Health Management, The First Affiliated Hospital of Anhui Medical University, Hefei, China; 3 Institute of Intelligent Machines, Hefei Institutes of Physical Science, Chinese Academy of Sciences, Hefei, China; 4 Department of Nursing, The Hefei Cancer Hospital, Chinese Academy of Sciences, Hefei, China

**Keywords:** aortic stiffness, cardiovascular risk stratification, carotid-femoral pulse wave velocity, insulin resistance, triglyceride-glucose index

## Abstract

**Background:**

Insulin resistance surrogate markers, including the triglyceride-glucose (TyG) index, TyG-BMI, and TG/HDL-C ratio, have been associated with aortic stiffness in high-risk populations. Their comparative associations and incremental discrimination in asymptomatic adults remain unclear. We therefore compared these markers and evaluated a simplified model for cfPWV-defined aortic stiffness.

**Methods:**

We conducted a cross-sectional study of 654 asymptomatic adults aged ≥18 years who underwent routine health examinations at two tertiary medical centers. After exclusions, 457 participants formed the modeling cohort and 101 formed the external validation cohort. Aortic stiffness was defined as cfPWV ≥10 m/s. We used multivariable logistic regression to estimate associations between the three surrogate markers and aortic stiffness. Restricted cubic spline regression assessed potential nonlinear associations. Incremental discrimination was evaluated using *Δ*AUC, NRI, and IDI. Six machine-learning models were compared, and the most interpretable model was evaluated in an independent institutional validation cohort.

**Results:**

Aortic stiffness was present in 32.8% (150/457) of the modeling cohort. After full adjustment, the TyG index was independently associated with aortic stiffness (per-SD OR = 3.44, *P* = 0.002), as were TG/HDL-C (OR = 3.11, *P* = 0.039) and, more weakly, TyG-BMI (OR = 1.52, *P* = 0.043). The model including TyG showed higher discrimination (*Δ*AUC = 0.014, DeLong *P* = 0.025) and reclassification (continuous NRI = 0.692 and IDI = 0.041; both *P* < 0.001) than the base model. A data-derived TyG cutoff of 8.68 was identified as a potential marker for exploratory risk stratification. The five-variable model, comprising age, MAP, diabetes mellitus, smoking, and TyG, yielded AUCs of 0.931 in the modeling cohort and 0.909 in the external validation cohort. Subgroup and sensitivity analyses showed similar patterns.

**Conclusion:**

Among the three surrogate markers, the TyG index showed the strongest adjusted association with aortic stiffness and the greatest incremental discrimination beyond conventional clinical variables. The five-variable model showed good discrimination in an independent institutional validation cohort. However, these cross-sectional findings support only potential risk-stratification value and do not establish temporal sequence, causality, or clinical utility; prospective studies are required to assess broader generalizability.

## GRAPHICAL ABSTRACT

**Figure:**
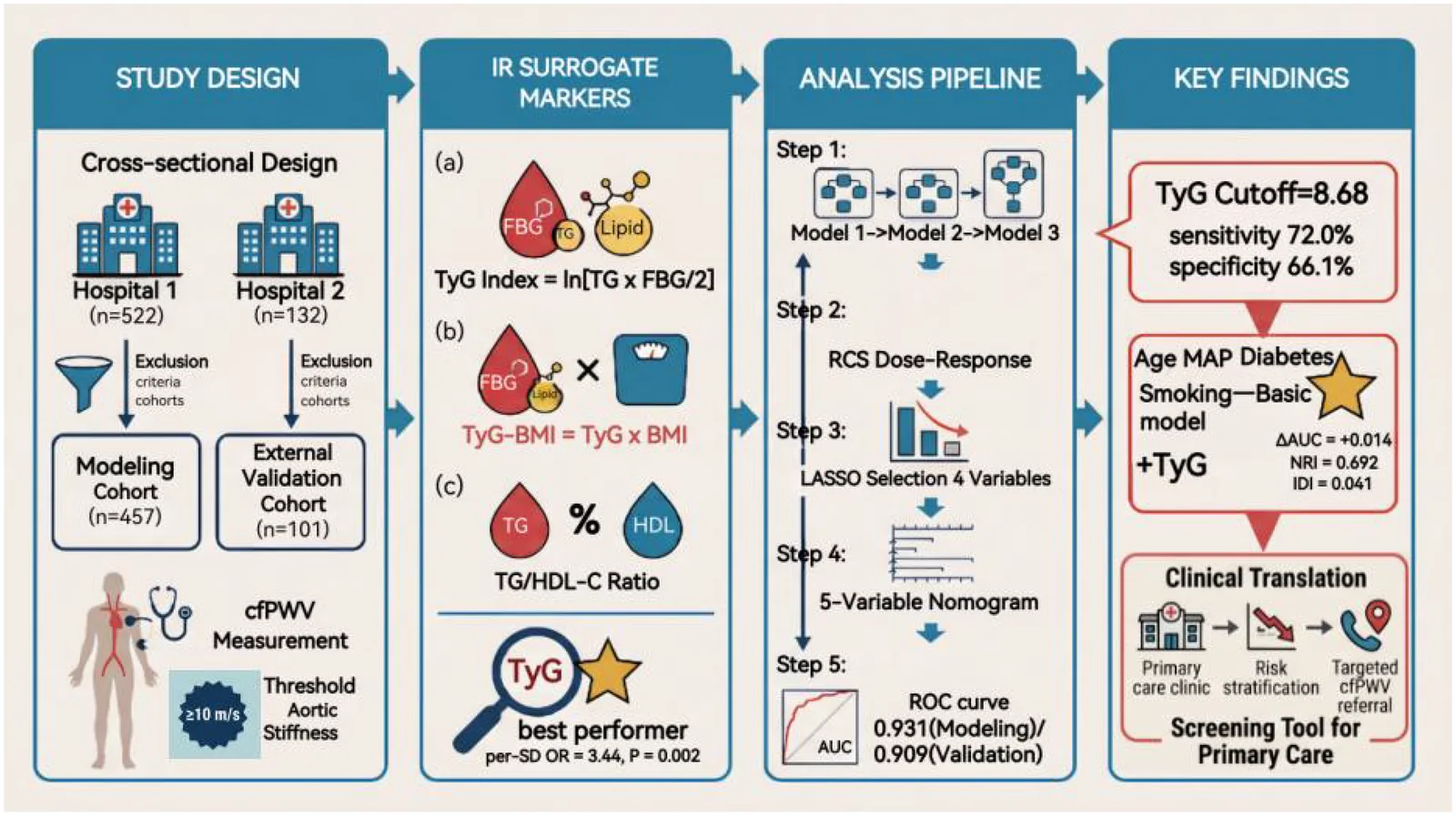

## 1 Introduction

Cardiovascular disease (CVD) represents a major global health burden, accounting for approximately 17.9 million deaths annually, or nearly one-third of all global deaths (1). Identifying subclinical markers of cardiovascular risk in asymptomatic populations is therefore a major public health priority. Aortic stiffness, which reflects reduced large-artery compliance, has been independently associated with subsequent major adverse cardiovascular events, including myocardial infarction, stroke, heart failure, and all-cause mortality, after accounting for conventional clinical variables such as age, hypertension, and hyperlipidemia (2).

Carotid-femoral pulse wave velocity (cfPWV) is the reference noninvasive measure of aortic stiffness. The 2018 European Society of Cardiology/European Society of Hypertension (ESC/ESH) Guidelines and the 2023 European Society of Hypertension (ESH) Guidelines recognize cfPWV as a marker of hypertension-mediated target-organ damage (3, 4). The 2023 ESH Guidelines recommend cfPWV ≥10 m/s as a threshold for aortic stiffness (4). However, cfPWV measurement requires specialized equipment and trained operators, limiting its use in routine screening (5). Accessible markers for identifying asymptomatic individuals who may benefit from confirmatory cfPWV assessment are therefore needed.

Insulin resistance (IR) has been associated with metabolic dysregulation, vascular dysfunction, and aortic stiffness (6). These relationships are consistent with pathophysiological hypotheses involving altered endothelial nitric oxide signaling, inflammation, oxidative stress, vascular smooth-muscle remodeling, collagen deposition, and elastin degradation (7). Although the hyperinsulinemic-euglycemic clamp is the reference method for quantifying IR, its cost and technical requirements limit clinical use (8). Several surrogate markers have therefore been developed. The triglyceride-glucose (TyG) index correlates with clamp-measured IR and has been associated with cardiometabolic outcomes (8, 9). TyG-BMI combines the TyG index with body mass index to incorporate adiposity (10). The TG/HDL-C ratio captures atherogenic dyslipidemia and is another accessible surrogate of IR and metabolic risk (11).

Conventional cardiovascular variables, including age, hypertension, hyperglycemia, and dyslipidemia, are incorporated into established approaches to aortic-stiffness risk stratification (2, 4, 12). However, the comparative performance of TyG, TyG-BMI, and TG/HDL-C has not been clearly established in asymptomatic adults undergoing routine health examinations. Previous studies have largely focused on patients with hypertension, diabetes, or established cardiovascular disease, limiting their applicability to primary-prevention populations (13). Few studies have directly compared these markers with cfPWV in the same cohort or developed simplified tools to prioritize confirmatory cfPWV assessment.

To address these gaps, we conducted a cross-sectional study with an independent institutional validation cohort. We aimed to (1) examine the adjusted associations of TyG, TyG-BMI, and TG/HDL-C with cfPWV-defined aortic stiffness; (2) compare their incremental discrimination beyond conventional clinical variables; and (3) develop and evaluate a simplified model to guide referral for confirmatory cfPWV assessment.

## 2 Methods

### 2.1 Study population

We conducted a cross-sectional study with external validation using data from two independent tertiary medical centers. The modeling cohort was recruited from the Health Management Center of the First Affiliated Hospital of Anhui Medical University. The external validation cohort was recruited from the Health Management Center of Hefei Cancer Hospital, Chinese Academy of Sciences. Between March and December 2024, consecutive adults aged ≥18 years undergoing routine health examinations were screened for eligibility. The modeling cohort included 231 women and 226 men. All participants completed cfPWV measurement and standardized laboratory blood tests.

Exclusion criteria were (1) a previous diagnosis of aortic aneurysm, aortic dissection, or another confirmed aortic disease; (2) severe peripheral arterial occlusive disease involving the carotid, upper-extremity, iliac, or femoral arteries that precluded cfPWV measurement; (3) severe hepatic or renal dysfunction or active malignancy; and (4) other disqualifying conditions according to investigator judgment, including pregnancy or inability to palpate the arterial pulse at measurement sites. After these criteria were applied, 457 participants from the First Affiliated Hospital of Anhui Medical University formed the modeling cohort, and 101 participants from Hefei Cancer Hospital formed the external validation cohort (Figure 1).

**Figure 1:**
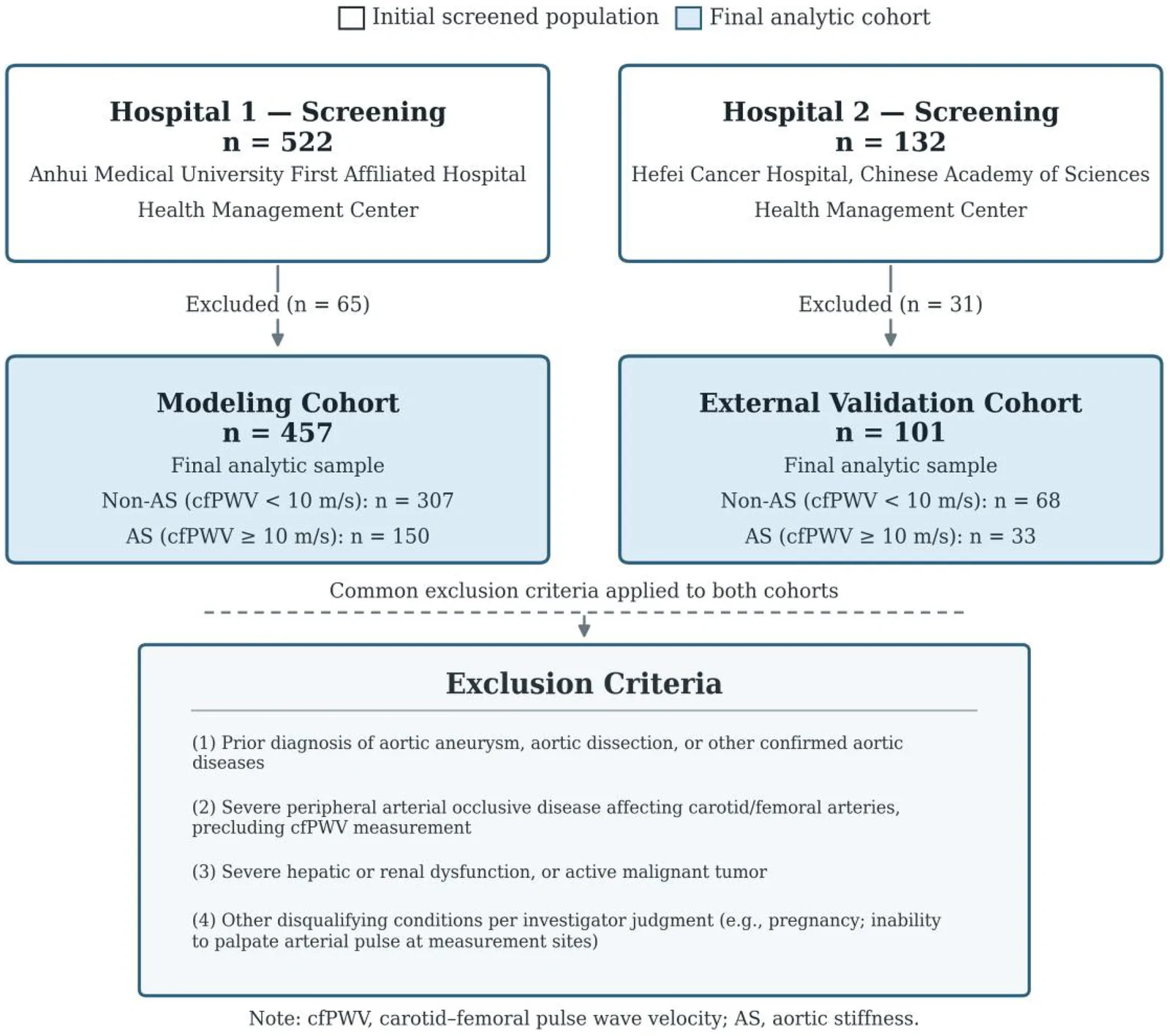
Flowchart of participant screening and enrollment. Boxes show screened and final analytic samples at the two centers; arrows indicate exclusions. AS, aortic stiffness; cfPWV, carotid-femoral pulse wave velocity.

All participants were asymptomatic adults, defined as having no self-reported history of cardiovascular disease and no cardiovascular symptoms, such as chest pain or intermittent claudication, at the time of examination. Participants with hypertension or diabetes who otherwise met the eligibility criteria were not excluded. Medical history and current antihypertensive and glucose-lowering medication use were recorded and included as covariates. Detailed information on drug class, dose, duration, adherence, lipid-lowering therapy, ethnicity, diet, and physical activity was not available in a sufficiently standardized form for analysis. The Ethics Committee of Hefei Institutes of Physical Science, Chinese Academy of Sciences, approved the study (YXLL−2023−46). The study followed the Declaration of Helsinki, and all participants provided written informed consent before enrollment.

### 2.2 Calculation of IR surrogate markers

The TyG index, BMI, TyG-BMI, and TG/HDL-C ratio were calculated using the following formulas:

$$\begin{array}{rl} & TyG\;=\;ln[triglycerides\;(mg/dL)\times fasting\;blood\;glucose\;(mg/dL)/2]\;(8)\\ & BMI\;(kg/m^{2})\;=\;weight\;(kg)\;/\;height\;{\left(m\right)}^{2}\\ & TyG-BMI\;=\;TyG\;\times \;BMI\;(10)\\ & TG/HDL-C\;=\;triglycerides\;(mg/dL)\;/\;HDL-C\;(mg/dL)\;(11) \end{array}$$

### 2.3 cfPWV measurement and definition of aortic stiffness

cfPWV was measured using the AtCor SphygmoCor XCEL device (AtCor Medical, Sydney, Australia). In a quiet, temperature-controlled room, after at least 10 min of supine rest, trained and certified technicians identified the pulse points at the sites of strongest pulsation over the right common carotid artery and right femoral artery (14). Participants were instructed to remain still and silent throughout the measurement. The direct carotid-femoral body-surface distance between the two maximal pulsation points was measured with a non-elastic tape measure and entered into the device software. The corrected path length was calculated as the direct distance (m) multiplied by 0.8, consistent with current European expert consensus recommendations (14). After obtaining at least 10 stable consecutive waveforms, cfPWV was calculated as: cfPWV (m/s) = corrected path length (m)/pulse transit time (s). Two consecutive measurements were taken, and the mean value was reported. If the difference between the two measurements exceeded 0.5 m/s, a third measurement was performed, and the median of the three measurements was used.

Aortic stiffness was defined as cfPWV ≥10 m/s, consistent with the threshold recommended by the 2023 European Society of Hypertension Guidelines (4). Previous research has also supported the use of this threshold in asymptomatic populations (15).

### 2.4 Covariates

We selected covariates *a priori* on the basis of clinical relevance and published literature (2, 4, 16) to control for potential confounding. These covariates included (i) demographic characteristics: age (years) and sex (female/male); (ii) lifestyle factors: smoking status (current or former regular smoking vs. never smoking) and alcohol consumption (≥1 alcoholic drink per week vs. none); (iii) clinical history: hypertension (SBP ≥140 mmHg and/or DBP ≥90 mmHg, self-reported diagnosis, or current use of antihypertensive medication) and diabetes mellitus (self-reported diagnosis, fasting blood glucose ≥7.0 mmol/L, or current use of glucose-lowering medication); (iv) physiological parameters: SBP, DBP, and MAP (mmHg); and (v) laboratory parameters: TC and LDL-C (mg/dL), ALT and AST (U/L), eGFR (mL/min/1.73 m^2^), creatinine (*μ*mol/L), uric acid (μmol/L), RBC count (×10^12^/L), hemoglobin (g/L), and platelet count (×10⁹/L). MAP was calculated as DBP + 1/3 × (SBP−DBP).

### 2.5 Statistical analysis

The sample size was determined by consecutive enrollment over a fixed period. To assess the adequacy of multivariable logistic regression, we calculated the events per variable (EPV). The modeling cohort included 150 events (cfPWV ≥10 m/s), resulting in an EPV of approximately 10 (150 events/15 candidate variables), consistent with the commonly cited minimum of 10 (17). LASSO selection and the lower-dimensional final model may reduce instability, but they do not eliminate the possibility of optimism. The independent institutional validation cohort therefore provides supportive evidence of transportability (18).

#### 2.5.1 Association analysis

We used multivariable-adjusted binary logistic regression to estimate associations between each IR surrogate marker (TyG index, TyG-BMI, and TG/HDL-C ratio) and aortic stiffness, and expressed the results as odds ratios (ORs) with 95% confidence intervals (CIs). Each exposure was categorized into quartiles (Q1–Q4), with the lowest quartile (Q1) serving as the reference group. Three nested models were constructed: Model 1 adjusted for demographic confounders (age and sex); Model 2 additionally adjusted for lifestyle factors and clinical history (smoking status, alcohol consumption, history of hypertension, history of diabetes mellitus, and use of antihypertensive and glucose-lowering medications); Model 3 further adjusted for comprehensive metabolic and hematological parameters (MAP, TC, LDL-C, ALT, AST, eGFR, creatinine, uric acid, RBC, hemoglobin, and platelet count). Linear trend tests were conducted by modeling the median value of each quartile as a continuous variable. To assess potential nonlinear exposure–outcome associations, restricted cubic spline (RCS) regression (19) was implemented using all adjustments from Model 3, with three knots placed at the 10th, 50th, and 90th percentiles (20). The *P* value for nonlinearity was obtained from the Wald test of the joint nonlinear spline terms, with *P* < 0.05 considered statistically significant. Because all variables were measured at the same examination, the ORs were interpreted as measures of association rather than causal effects.

#### 2.5.2 Subgroup and interaction analyses

We assessed whether the associations were consistent across prespecified subgroups defined by age (<60 vs. ≥60 years), sex, hypertension, diabetes mellitus, smoking, alcohol consumption, antihypertensive medication use, and glucose-lowering medication use. Within each stratum, ORs for each IR surrogate marker were estimated using the fully adjusted Model 3. Multiplicative interaction terms between each stratification variable and the corresponding IR marker were used to test effect modification. An interaction *P* < 0.05 was considered evidence of statistically significant effect modification.

#### 2.5.3 Variable selection

LASSO regression (18) was applied in the modeling cohort to identify a parsimonious set of candidate variables associated with aortic stiffness. IR-related indices and their metabolic components (triglycerides, fasting blood glucose, and BMI) were excluded at this step to prevent collinearity with the IR surrogate markers subsequently added (21). SBP and DBP were also excluded to prevent collinearity with MAP, leaving 15 prespecified candidate clinical variables for LASSO selection. The optimal regularization parameter (*λ*) was determined via 10-fold cross-validation using the one-standard-error rule (*λ*₁ₛₑ), retaining only variables with nonzero LASSO coefficients to form the base model.

#### 2.5.4 Discriminative ability and incremental model performance

Using the base model identified by LASSO, each IR surrogate marker (TyG index, TyG-BMI, TG/HDL-C) was added individually to construct three extended models. Discriminative ability was assessed using the area under the receiver operating characteristic curve (AUC), with 95% CIs estimated by 1,000 bootstrap resamples. Differences in AUC between each enhanced model and the base model were tested using the DeLong method. Incremental model performance was quantified using the category-free (continuous) net reclassification improvement (NRI) and integrated discrimination improvement (IDI), and statistical significance was assessed using 1,000 bootstrap resamples. Based on the comprehensive evaluation of *Δ*AUC, NRI, and IDI, the IR surrogate marker with the largest *Δ*AUC and statistically significant NRI and IDI was selected for inclusion in the final risk-stratification model, and the optimal discriminatory threshold was subsequently determined by maximizing the Youden index.

#### 2.5.5 Risk-stratification model construction and validation

We evaluated six machine-learning algorithms in the modeling cohort: logistic regression, random forest, support vector machine (SVM), XGBoost, decision tree, and naive Bayes. All models incorporated the covariates selected by LASSO regression and the best-performing IR surrogate marker. Grid search with 10-fold cross-validation was used to select optimal hyperparameters. Discrimination was assessed using AUC, and models were compared using the DeLong test. The final model was selected by balancing discrimination and clinical interpretability. External validation was performed in an independent cohort from Hefei Cancer Hospital, Chinese Academy of Sciences (*n* = 101), which was not involved in model development. External performance was evaluated using AUC, calibration curves, the Hosmer-Lemeshow goodness-of-fit test, and decision-curve analysis (22).

#### 2.5.6 Sensitivity analysis

We conducted sensitivity analyses to assess the robustness of the primary findings. First, the selected risk-stratification model underwent 10-fold cross-validation in the modeling cohort (*n* = 457). The cohort was partitioned into 10 subsets; in each iteration, nine subsets were used for training and the remaining subset for validation. Performance was summarized as the mean AUC across the 10 folds. Second, the primary analyses were repeated after excluding participants with hypertension (*n* = 299), diabetes mellitus (*n* = 406), or both conditions (*n* = 284).

#### 2.5.7 Statistical software

Continuous variables were reported as mean ± standard deviation (SD) or median [interquartile range (IQR)], according to their distribution, and were compared using Student's t-test or the Mann–Whitney U test. Categorical variables were reported as counts (percentages) and compared using the chi-square test or Fisher's exact test. All tests were two-sided, and *P* < 0.05 was considered statistically significant. Logistic regression, restricted cubic spline regression, and nomogram construction were performed using R version 4.3.0 [R Foundation for Statistical Computing, Vienna, Austria; rms package (19)]; decision-curve analysis used the dcurves package. LASSO regression, ROC analysis, 10-fold cross-validation, random forest, SVM, decision tree, and naive Bayes were implemented in Python version 3.11 using scikit-learn (23). XGBoost used the xgboost package. NRI and IDI were calculated using custom Python functions.

## 3 Results

### 3.1 Study population and baseline characteristics

The modeling cohort included 457 participants: 226 men (49.5%) and 231 women (50.5%). Participants ranged in age from 22 to 91 years, with a mean age of 50.7 ± 16.3 years. Aortic stiffness was present in 150 participants (32.8%), whereas 307 participants (67.2%) were classified as non-AS.

Compared with participants without aortic stiffness, those with aortic stiffness were older and had higher BMI, SBP, DBP, fasting blood glucose, triglycerides, LDL-C, TC, ALT, AST, TyG, TyG-BMI, and TG/HDL-C, together with lower eGFR and platelet counts (all *P* < 0.05). They also had higher frequencies of alcohol consumption, hypertension, diabetes, antihypertensive medication use, and glucose-lowering medication use (all *P* < 0.05). Sex, smoking, creatinine, uric acid, HDL-C, RBC count, and hemoglobin did not differ significantly between groups (all *P* > 0.05). Table 1 and Supplementary Table S1 provide detailed characteristics; Supplementary Tables S2–S4 present quartile-stratified characteristics.

**Table 1:** Baseline characteristics of participants according to aortic stiffness status.

Characteristic	Total (*n* = 457)	Non-AS (cfPWV <10 m/s) (*n* = 307)	AS (cfPWV ≥ 10 m/s) (*n* = 150)	*P* value
Age (years)	50.7 ± 16.3	44.3 ± 14.4	64.3 ± 10.8	<0.001
Sex (Male), *n* (%)	226 (49.5%)	150 (48.9%)	76 (50.7%)	0.388
BMI (kg/m^2^)	23.7 ± 3.6	23.4 ± 3.2	24.4 ± 4.1	0.007
SBP (mmHg)	124.9 ± 15.3	118.9 ± 12.1	137.7 ± 13.3	<0.001
DBP (mmHg)	76.3 ± 10.0	73.5 ± 8.9	82.4 ± 9.5	<0.001
MAP (mmHg)	92.5 ± 10.9	88.7 ± 9.4	100.3 ± 9.7	<0.001
Hypertension, *n* (%)	141 (30.9%)	47 (15.3%)	94 (62.7%)	<0.001
Antihypertensive medication, *n* (%)	74 (16.2%)	22 (7.2%)	52 (34.7%)	<0.001
Diabetes mellitus, *n* (%)	36 (7.9%)	9 (2.9%)	27 (18.0%)	<0.001
Antidiabetic medication, *n* (%)	34 (7.4%)	12 (3.9%)	22 (14.7%)	<0.001
Smoking, *n* (%)	65 (14.2%)	43 (14.0%)	22 (14.7%)	0.692
Alcohol consumption, *n* (%)	69 (15.1%)	35 (11.4%)	34 (22.7%)	<0.001
TG (mg/dL)	111.6 [79.7, 161.2]	103.6 [75.1, 147.2]	137.3 [93.0, 194.0]	<0.001
TC (mg/dL)	193.8 ± 40.2	189.4 ± 38.6	203.2 ± 42.1	<0.001
HDL-C (mg/dL)	54.5 [47.4, 62.4]	53.0 [47.2, 62.0]	55.7 [48.7, 62.6]	0.164
LDL-C (mg/dL)	128.6 ± 30.4	126.1 ± 29.9	133.9 ± 31.0	0.011
FBG (mg/dL)	94.4 [88.3, 106.7]	92.1 [87.0, 97.4]	112.8 [100.0, 125.8]	<0.001
TyG Index	8.6 [8.2, 9.0]	8.5 [8.2, 8.9]	8.9 [8.6, 9.3]	<0.001
TyG-BMI	201.8 [177.9, 229.4]	196.5 [174.1, 223.3]	214.9 [193.8, 244.2]	<0.001
TG/HDL-C	2.2 [1.4, 3.2]	2.0 [1.3, 2.9]	2.4 [1.5, 4.0]	<0.001
ALT (U/L)	19.5 [14.0, 27.7]	18.9 [12.9, 27.0]	22.0 [16.3, 28.0]	0.006
AST (U/L)	22.0 [19.0, 26.0]	21.7 [18.0, 25.7]	23.0 [21.0, 26.0]	<0.001
eGFR (mL/min/1.73m^2^)	105.0 ± 16.6	110.3 ± 14.7	93.6 ± 14.5	<0.001
Creatinine (μmol/L)	66.9 [58.0, 78.2]	66.3 [57.2, 77.8]	67.6 [59.6, 79.9]	0.107
Uric acid (μmol/L)	344.1 ± 93.4	344.8 ± 98.0	342.5 ± 82.9	0.810
RBC (×10^12^/L)	4.7 ± 0.5	4.7 ± 0.5	4.6 ± 0.4	0.557
HGB (g/L)	140.4 ± 14.8	140.0 ± 15.2	141.1 ± 13.9	0.478
Platelet (×10⁹/L)	219.0 [180.0, 257.0]	222.0 [183.5, 263.5]	213.0 [172.0, 245.0]	0.042
cfPWV (m/s)	9.2 [8.3, 10.5]	8.7 [7.9, 9.3]	11.2 [10.6, 12.3]	<0.001

AS, aortic stiffness; BMI, body mass index; SBP, systolic blood pressure; DBP, diastolic blood pressure; MAP, mean arterial pressure; TG, triglycerides; TC, total cholesterol; HDL-C, high-density lipoprotein cholesterol; LDL-C, low-density lipoprotein cholesterol; FBG, fasting blood glucose; TyG, triglyceride-glucose; ALT, alanine aminotransferase; AST, aspartate aminotransferase; eGFR, estimated glomerular filtration rate; RBC, red blood cells; HGB, hemoglobin; PLT, platelets; cfPWV, carotid-femoral pulse wave velocity.

### 3.2 Associations of IR markers with aortic stiffness

#### 3.2.1 Multivariable logistic regression

Associations between the three surrogate markers and aortic stiffness, assessed using quartile-based multivariable logistic regression, are presented in Table 2. In the fully adjusted Model 3, the TyG index was independently associated with aortic stiffness (per-SD OR = 3.44, 95% CI: 2.03–5.82, *P* = 0.002), with a significant linear trend across quartiles (P for trend = 0.010). TG/HDL-C was also associated with aortic stiffness (per-SD OR = 3.11, 95% CI: 1.90–5.09, *P* = 0.039). TyG-BMI showed a weaker association (per-SD OR = 1.52, 95% CI: 1.03–2.25, *P* = 0.043), and its linear trend was not statistically significant (P for trend = 0.086). None of the Q4-versus-Q1 comparisons reached statistical significance after full adjustment (all *P* > 0.05).

**Table 2:** Associations of TyG index, TyG-BMI, and TG/HDL-C with aortic stiffness (logistic regression, Q1 as reference).

Variables	Model 1 OR (95% CI)	Model 2 OR (95% CI)	Model 3 OR (95% CI)	*P* value (Model 3)	*P* for trend
TyG Index					
Q1 (Reference)	Reference	Reference	Reference	—	
Q2 vs Q1	1.54 (0.65–3.65)	1.76 (0.73–4.26)	0.93 (0.26–3.25)	0.904	
Q3 vs Q1	3.95 (1.74–8.97)**	3.95 (1.66–9.42)*	5.45 (1.31–22.64)*	0.020	
Q4 vs Q1	6.23 (2.72–14.27)**	5.70 (2.45–13.25)**	2.79 (0.83–9.31)	0.096	
Per 1 SD increase	2.51 (1.84–3.43)**	2.36 (1.71–3.25)**	3.44 (2.03–5.82)**	0.002	
*P for trend*					*0*.*010*
TyG-BMI					
Q1 (Reference)	Reference	Reference	Reference	—	
Q2 vs Q1	1.33 (0.59–2.98)	1.48 (0.65–3.38)	1.19 (0.32–4.42)	0.792	
Q3 vs Q1	1.91 (0.88–4.16)	2.04 (0.90–4.60)	1.46 (0.36–6.04)	0.597	
Q4 vs Q1	3.37 (1.49–7.61)*	2.58 (1.10–6.03)*	3.38 (0.90–12.61)	0.070	
Per 1 SD increase	1.82 (1.36–2.45)**	1.76 (1.29–2.40)**	1.52 (1.03–2.25)*	0.043	
*P for trend*					*0*.*086*
TG/HDL-C					
Q1 (Reference)	Reference	Reference	Reference	—	
Q2 vs Q1	1.16 (0.53–2.52)	1.28 (0.57–2.84)	1.66 (0.50–5.55)	0.409	
Q3 vs Q1	1.17 (0.53–2.57)	1.28 (0.58–2.86)	2.74 (0.69–10.85)	0.152	
Q4 vs Q1	3.20 (1.48–6.94)*	2.83 (1.28–6.28)*	2.65 (0.80–8.78)	0.112	
Per 1 SD increase	1.94 (1.39–2.69)**	1.85 (1.30–2.62)**	3.11 (1.90–5.09)*	0.039	
*P for trend*					*0*.*079*

OR, odds ratio; CI, confidence interval; SD, standard deviation. Model 1 adjusted for demographic confounders (age and sex); Model 2 additionally adjusted for lifestyle factors and clinical history (smoking status, alcohol consumption, history of hypertension, history of diabetes mellitus, and use of antihypertensive and glucose-lowering medications); Model 3 further adjusted for comprehensive metabolic and hematological parameters (MAP, TC, LDL-C, ALT, AST, eGFR, creatinine, uric acid, RBC, hemoglobin, and platelet count). *, **: *P* < 0.05 and *P* < 0.01, respectively. *P* values shown are from Model 3.

#### 3.2.2 Subgroup analysis

In stratified analyses, all three surrogate markers showed generally consistent positive associations with aortic stiffness across the prespecified subgroups (Table 3). In the overall cohort, each unit increase in TyG was associated with higher odds of aortic stiffness (OR = 3.91, 95% CI: 1.87–8.15, *P* < 0.001). TyG-BMI and TG/HDL-C were also positively associated with aortic stiffness. No statistically significant interactions were detected between any marker and the prespecified stratification variables (all interaction *P* > 0.05).

**Table 3:** Subgroup analysis of TyG index, TyG-BMI, and TG/HDL-C ratio with aortic stiffness.

Subgroup	n	TyG index		TyG-BMI		TG/HDL-C ratio		P for interaction		
		OR (95% CI)	P value	OR (95% CI)	P value	OR (95% CI)	*P* value	TyG index	TyG-BMI	TG/HDL-C
Overall	457	3.91 (1.87–8.15)	<0.001	1.01 (1.00–1.03)	0.014	1.28 (1.05–1.56)	0.014	-	-	-
Age (years)								0.221	0.865	0.956
<60	315	2.16 (0.88–5.28)	0.092	1.02 (1.00–1.03)	0.041	1.25 (0.95–1.66)	0.114			
≥60	142	24.40 (2.43–244.57)	0.007	1.02 (1.00–1.04)	0.021	1.51 (0.86–2.66)	0.152			
Sex								0.715	0.201	0.712
Female	231	4.57 (1.35–15.51)	0.015	1.00 (0.99–1.02)	0.573	1.29 (0.85–1.93)	0.228			
Male	226	2.89 (1.06–7.86)	0.038	1.03 (1.01–1.05)	0.013	1.22 (0.98–1.52)	0.079			
Hypertension								0.116	0.171	0.110
No	316	5.71 (1.93–16.93)	0.002	1.02 (1.00–1.03)	0.017	1.35 (0.99–1.84)	0.057			
Yes	141	2.12 (0.58–7.74)	0.257	1.01 (0.99–1.03)	0.406	1.05 (0.71–1.56)	0.793			
Diabetes Mellitus								0.969	0.991	0.736
No	421	3.99 (1.78–8.93)	< 0.001	1.01 (1.00–1.03)	0.015	1.26 (1.03–1.54)	0.024			
Yes	36	11.65 (1.20–113.38)	0.034	1.04 (0.96–1.13)	0.365	2.65 (0.94–7.47)	0.066			
Smoking								0.536	0.524	0.857
No	392	4.63 (1.90–11.29)	< 0.001	1.02 (1.00–1.03)	0.018	1.35 (1.05–1.72)	0.018			
Yes	65	2.11 (0.57–7.87)	0.266	1.00 (0.98–1.02)	0.904	1.16 (0.92–1.47)	0.207			
Alcohol consumption								0.177	0.105	0.192
No	370	3.28 (1.39–7.74)	0.007	1.01 (0.99–1.02)	0.279	1.21 (0.96–1.52)	0.111			
Yes	69	3.42 (0.54–21.69)	0.191	1.05 (1.01–1.11)	0.030	1.24 (0.95–1.61)	0.118			
Antihypertensive medications								0.164	0.492	0.171
No	383	3.07 (1.35–6.96)	0.007	1.01 (1.00–1.03)	0.034	1.20 (0.98–1.47)	0.075			
Yes	74	18.21 (1.78–186.24)	0.014	1.02 (0.99–1.05)	0.152	2.72 (0.42–17.54)	0.291			
Antidiabetic medications								0.652	0.453	0.997
No	423	3.71 (1.73–7.96)	<0.001	1.01 (1.00–1.03)	0.017	1.26 (1.03–1.54)	0.023			
Yes	34	8.86 (1.04–75.36)	0.046	1.00 (0.94–1.05)	0.914	6.97 (0.44–110.42)	0.168			

OR, odds ratio; CI, confidence interval. All ORs in this table are expressed per unit increment of the respective index (not per SD); all subgroup estimates used the fully adjusted Model 3. Interaction *P* values are derived from multiplicative interaction terms between stratification variables and the corresponding index.

#### 3.2.3 Nonlinear exposure–outcome associations

Restricted cubic spline regression was used to characterize potential nonlinear associations between each surrogate marker and aortic stiffness. In fully adjusted Model 3, TyG showed a significant overall association, with no statistically significant evidence of nonlinearity (P for overall association = 0.003; P for nonlinearity = 0.069). TyG-BMI showed both an overall association and evidence of nonlinearity (*P* = 0.004 and P for nonlinearity = 0.022). TG/HDL-C showed an overall association without evidence of nonlinearity (*P* = 0.045 and P for nonlinearity = 0.162). Figure 2 shows the corresponding curves.

**Figure 2:**
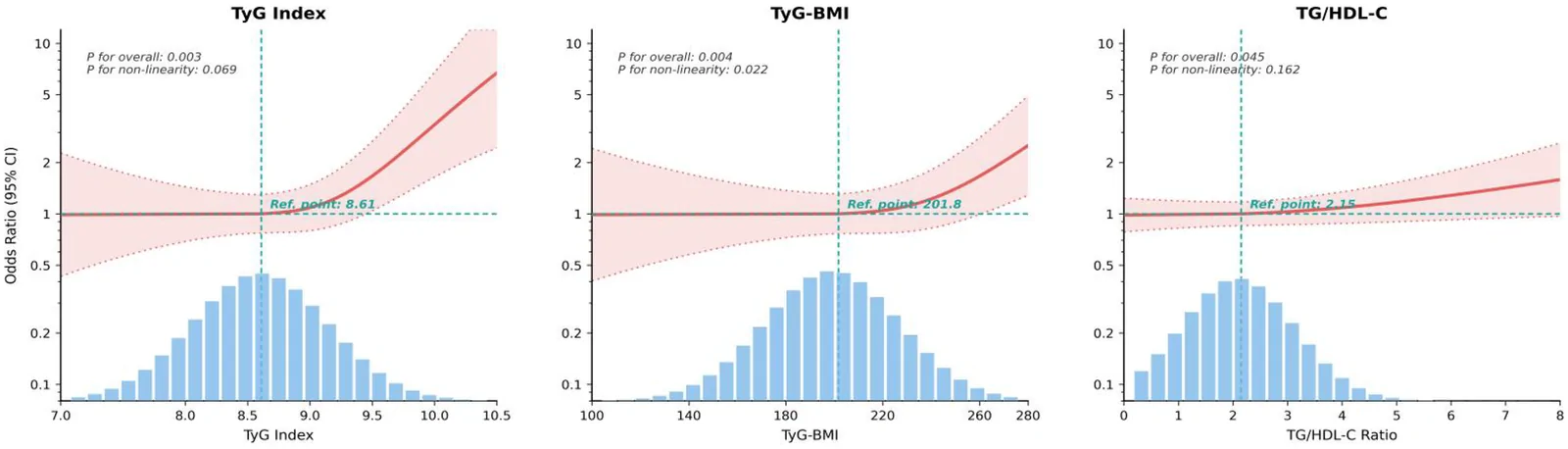
Restricted cubic spline analyses of TyG index, TyG-BMI, and TG/HDL-C in relation to aortic stiffness in fully adjusted model 3. The left, middle, and right panels show TyG index, TyG-BMI, and TG/HDL-C. Solid curves represent adjusted odds ratios, shaded areas represent 95% confidence intervals, and dashed lines indicate the reference odds ratio and reference values.

### 3.3 Variable selection using LASSO regression

LASSO-penalized logistic regression was applied to 15 prespecified candidate clinical variables using 10-fold cross-validation and binomial deviance. The *λ*1se criterion retained four variables for the base model: age, MAP, diabetes mellitus, and smoking (Table 4 and Figure 3). Their standardized coefficients were 1.193, 0.950, 0.204, and 0.077, respectively. All retained variables had variance inflation factors below 2.

**Figure 3:**
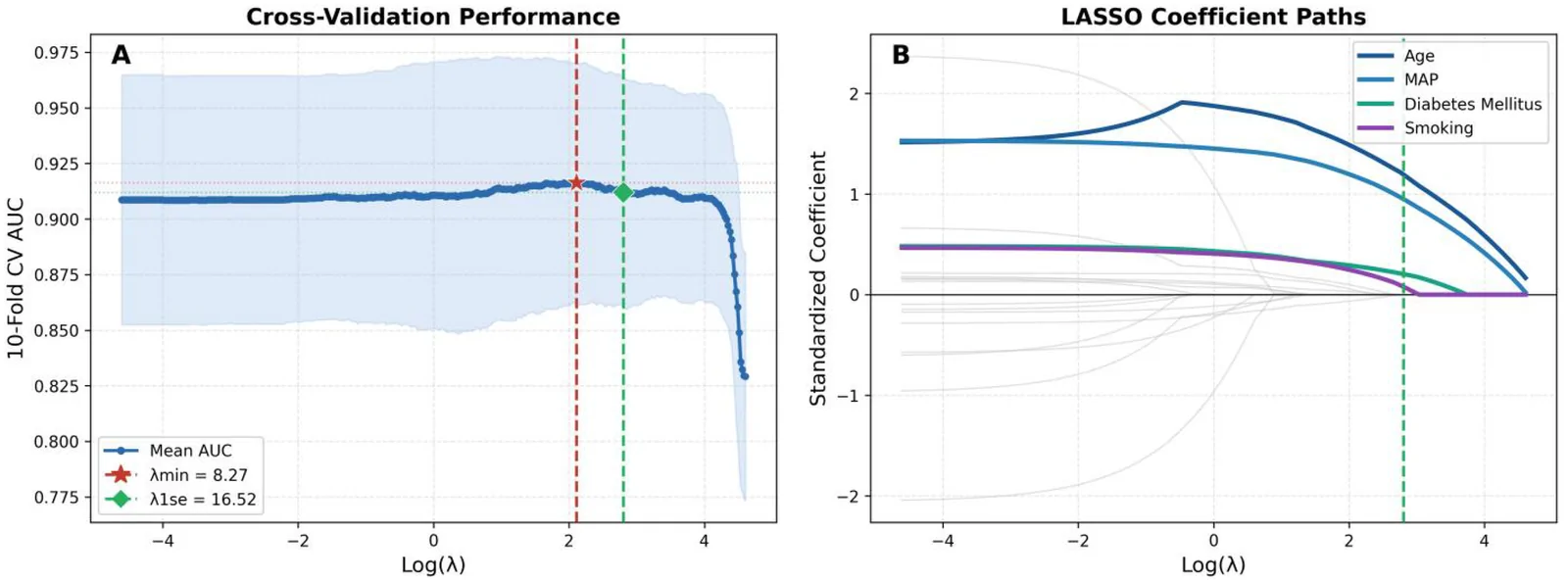
LASSO variable selection for the base risk-stratification model. **(A)** Ten-fold cross-validated AUC according to log(*λ*); dashed lines indicate *λ*min and *λ*1se. **(B)** LASSO coefficient paths; colored paths identify the variables retained at *λ*1se.

**Table 4:** Variables retained by LASSO using the *λ*1se criterion in the base model.

Variable	Standardized Coefficient	VIF
Age	1.193	1.13
MAP	0.950	1.09
Diabetes mellitus	0.204	1.08
Smoking	0.077	1.02

#### 3.3.1 Incremental discriminative performance of IR markers

The base model, comprising age, MAP, diabetes mellitus, and smoking, yielded an AUC of 0.917 (95% CI: 0.890–0.943). The corresponding extended models including TyG, TyG-BMI, or TG/HDL-C showed AUCs of 0.931, 0.919, and 0.930, respectively. The corresponding *Δ*AUC values were 0.014, 0.002, and 0.013. DeLong tests were significant for TyG and TG/HDL-C (*P* = 0.025 and *P* = 0.032, respectively) but not for TyG-BMI (*P* = 0.682). All three enhanced models showed significant continuous NRI, whereas IDI was significant for TyG and TG/HDL-C but not for TyG-BMI (Table 5).

**Table 5:** Incremental discriminative performance of IR markers added to the base model.

Model	AUC (95% CI)	*Δ*AUC	DeLong P	NRI	NRI P	IDI	IDI P
Basic Model (Age + MAP + Diabetes Mellitus + Smoking)	0.917 (0.890–0.943)	-	-	-	-	-	-
Basic + TyG Index	0.931 (0.920–0.961)	+0.014	0.025	0.692	<0.001	0.041	<0.001
Basic + TyG-BMI	0.919 (0.892–0.944)	+0.002	0.682	0.454	<0.001	0.006	0.364
Basic + TG/HDL-C	0.930 (0.905–0.953)	+0.013	0.032	0.564	<0.001	0.034	0.005

AUC, area under the ROC curve; CI, confidence interval; *Δ*AUC, change in AUC relative to the base model; NRI, net reclassification improvement (category-free); IDI, integrated discrimination improvement, *P* < 0.05 indicates significance. AUC 95% CIs were estimated by 1,000 bootstrap resamples; DeLong test was used for between-model AUC comparisons.

### 3.4 Optimal cutoff value

The Youden index identified a TyG cutoff of 8.68 in the modeling cohort (*n* = 457), with a sensitivity of 0.720, specificity of 0.661, PPV of 0.509, and NPV of 0.829. Applying the same candidate threshold to the external validation cohort (*n* = 101) yielded a sensitivity of 0.636, specificity of 0.632, PPV of 0.457, NPV of 0.782, and a detection rate of 63.6% (21/33) (Table 6).

**Table 6:** Diagnostic performance of the optimal TyG index cutoff value for discriminating prevalent aortic stiffness.

Cohort	AUC (95% CI)	Threshold	Sensitivity	Specificity	PPV	NPV	Detection Rate
Modeling cohort (*n* = 457)	0.752 (0.704–0.797)	8.68	0.720	0.661	0.509	0.829	0.236
Validation cohort (*n* = 101)	0.694 (0.578–0.802)	8.68	0.636	0.632	0.457	0.782	0.208

Detection rate = proportion of participants with TyG ≥8.68 in whom aortic stiffness was detected. PPV, positive predictive value; NPV, negative predictive value. The cutoff value was determined using the Youden index (maximizing sensitivity + specificity−1).

### 3.5 Model comparison

After grid-search hyperparameter optimization in the modeling cohort, six machine-learning models were compared (Figure 4 and Table 7; Supplementary Table S5 lists the optimal hyperparameters). XGBoost had the highest AUC (0.938), but its discrimination did not differ significantly from that of logistic regression (AUC = 0.931; DeLong *P* = 0.462; Table 8). Logistic regression was selected because it provided comparable discrimination with greater interpretability.

**Figure 4:**
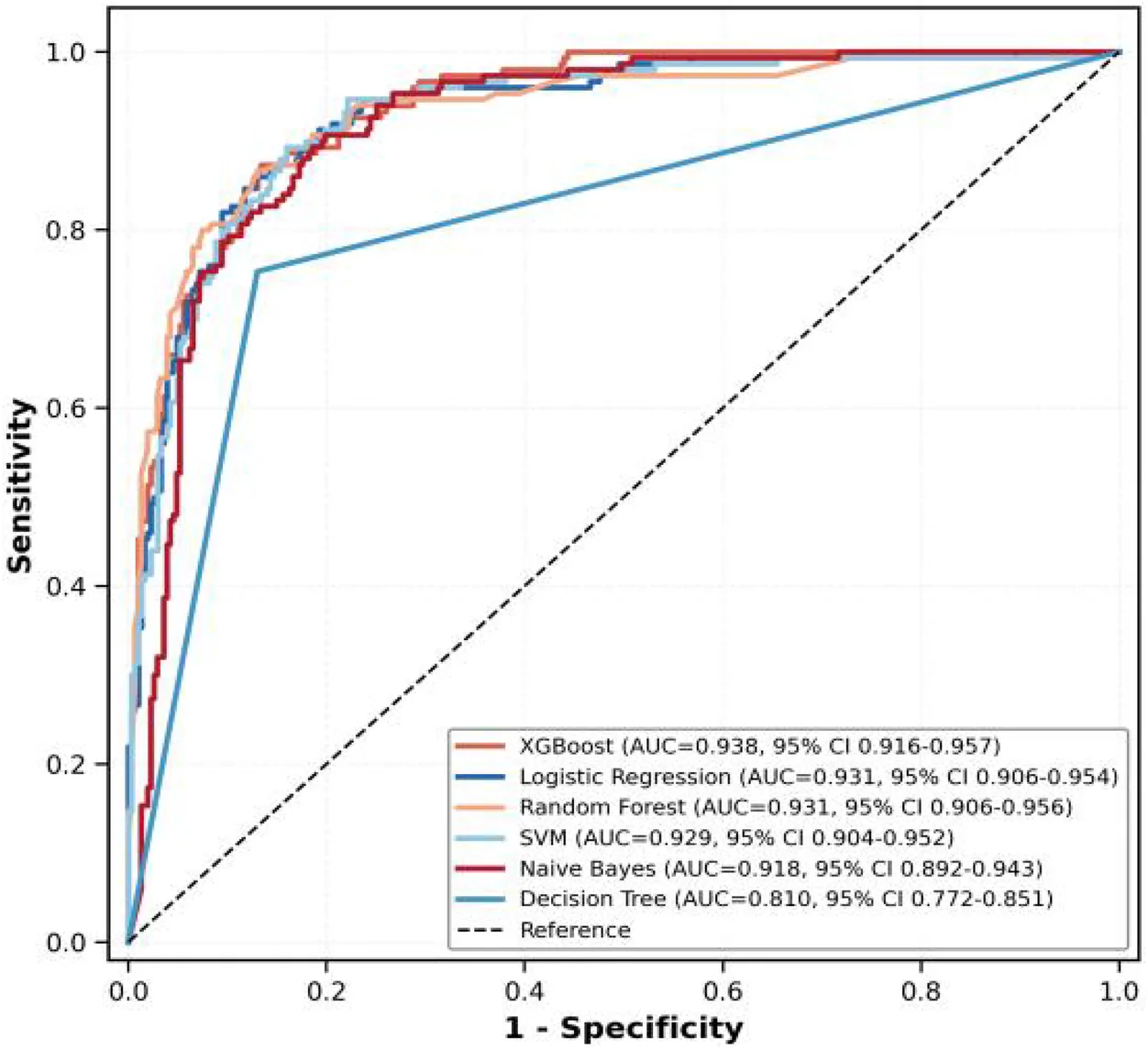
ROC curves comparing six machine-learning models. The legend identifies each model and its AUC; the diagonal line represents chance performance.

**Table 7:** Performance metrics of the six machine learning models.

Model	AUC	Sensitivity	Specificity	PPV	NPV	Accuracy
Logistic Regression	0.931	0.867	0.863	0.756	0.930	0.864
XGBoost	0.938	0.860	0.876	0.772	0.928	0.871
Decision Tree	0.810	0.753	0.870	0.739	0.878	0.832
Random Forest	0.931	0.867	0.870	0.765	0.930	0.869
SVM	0.929	0.807	0.876	0.761	0.903	0.853
Naive Bayes	0.918	0.907	0.801	0.690	0.946	0.836

**Table 8:** *P* values from deLong test for pairwise AUC comparisons.

Model	Logistic Regression	XGBoost	Decision Tree	Random Forest	SVM	Naive Bayes
Logistic Regression	–	0.462	＜0.001	0.998	0.760	0.066
XGBoost		–	＜0.001	0.273	0.283	0.067
Decision Tree			–	＜0.001	＜0.001	＜0.001
Random Forest				–	0.822	0.246
SVM					–	0.134
Naive Bayes						–

### 3.6 Nomogram-based risk-stratification model construction

The final logistic regression model included age, MAP, diabetes mellitus, smoking, and TyG. A graphical nomogram was constructed from the modeling cohort to support individualized risk estimation. For example, a 50-year-old nonsmoker with MAP = 90 mmHg, diabetes mellitus, and TyG = 10.5 accumulated approximately 136 points, corresponding to an estimated aortic-stiffness probability of approximately 70% (Figure 5). Although the underlying five-variable equation was evaluated in the institutional validation cohort, the graphical nomogram was not separately externally validated or recalibrated.

**Figure 5:**
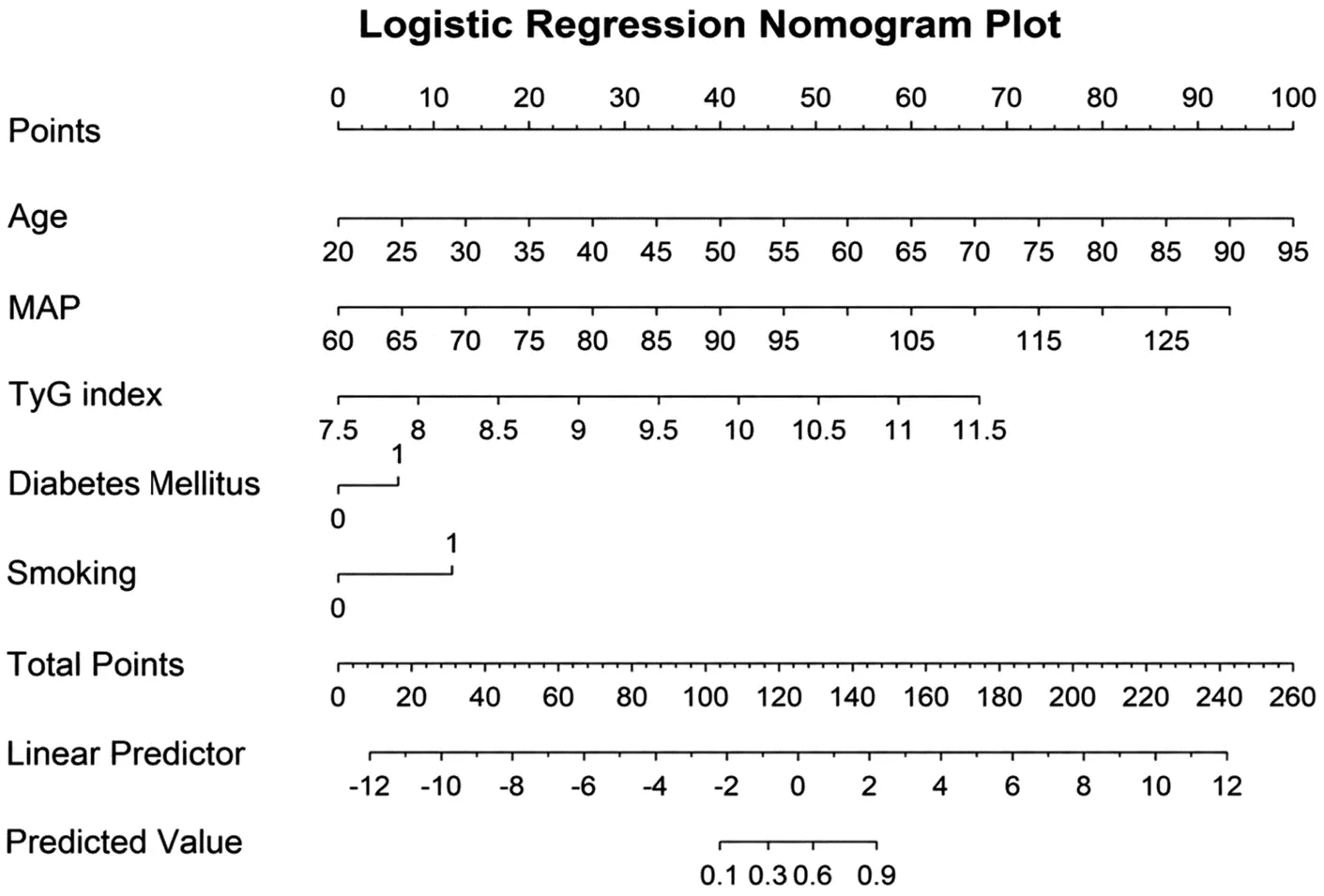
Nomogram for individualized probability estimation of prevalent aortic stiffness. Model-variable scales assign points that are summed and mapped to the linear predictor and model-estimated probability. The nomogram was derived from the modeling cohort and was not separately externally validated or recalibrated.

### 3.7 Model performance and external validation

The final five-variable model, comprising age, MAP, diabetes mellitus, smoking, and TyG, achieved an AUC of 0.931 (95% CI: 0.920–0.961; Brier score = 0.090) in the modeling cohort and 0.909 (95% CI: 0.866–0.973; Brier score = 0.119) in the external validation cohort. Calibration curves showed agreement between model-estimated and observed probabilities in both cohorts. Decision-curve analysis suggested estimated net benefit over treat-all and treat-none strategies across a range of threshold probabilities within these datasets (Figure 6). However, discrimination and statistical significance do not establish clinical utility. TyG alone showed only moderate discrimination (AUC = 0.752 in the modeling cohort and 0.694 in the external validation cohort), with a validation PPV of 45.7% and NPV of 78.2%. The standalone threshold cannot replace confirmatory cfPWV measurement. Moreover, adding TyG to the base model was associated with only a modest AUC increase (*Δ*AUC = 0.014). Prospective impact studies are needed to determine whether model-guided referral improves clinical decisions or patient outcomes.

**Figure 6:**
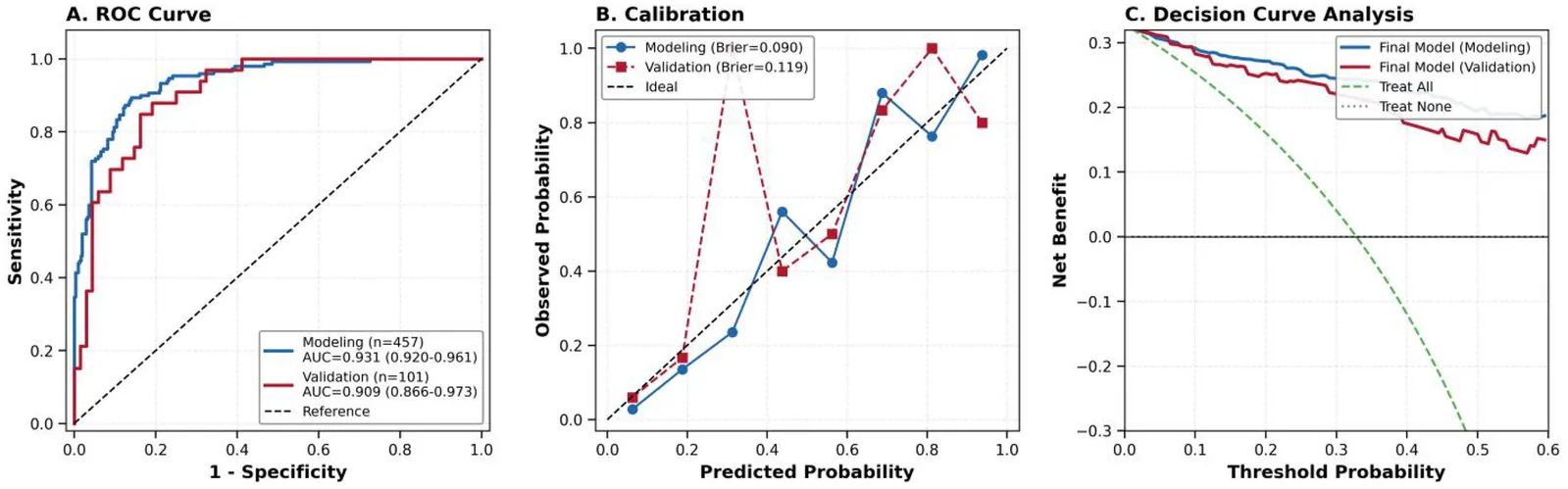
Performance of the five-variable logistic regression model in the modeling and external validation cohorts. **(A)** ROC curves and AUCs. **(B)** Calibration curves comparing model-estimated and observed probabilities. **(C)** Decision curves showing estimated net benefit across threshold probabilities; cohorts are identified in the legend.

### 3.8 Sensitivity analysis

Sensitivity analyses supported the primary findings. After exclusion of participants with hypertension, the TyG association strengthened (OR = 2.65, *P* = 0.003), TyG-BMI remained significant (OR = 1.88, *P* = 0.024), and TG/HDL-C was no longer statistically significant (OR = 1.79, *P* = 0.097). Similar patterns were observed after exclusion of diabetes and after excluding both conditions (Supplementary Table S6). In the modeling cohort, 10-fold cross-validation yielded a mean AUC of 0.948, with sensitivity of 0.829, specificity of 0.914, PPV of 0.833, and NPV of 0.920. The mean Brier score was 0.085 (Supplementary Table S7 and Supplementary Figure S1).

## 4 Discussion

In this cross-sectional study of Chinese adults undergoing routine health examinations, we compared the associations of TyG, TyG-BMI, and TG/HDL-C with cfPWV-defined aortic stiffness. TyG showed the strongest adjusted association among the three markers. A candidate TyG cutoff of 8.68 showed moderate discrimination in the institutional validation cohort, and the five-variable model showed good discrimination in both cohorts. However, all clinical and medication variables, TyG, and cfPWV were assessed at the same examination. We therefore could not determine whether the clinical variables preceded TyG elevation or whether TyG elevation preceded aortic stiffening. The cross-sectional design does not support causal inference, and reverse causation and time-varying or unmeasured confounding remain possible. Longitudinal studies with repeated TyG and cfPWV measurements are required to establish temporal ordering.

cfPWV is the reference noninvasive measure of aortic stiffness and is used to characterize functional vascular abnormalities that may be observed before overt structural changes (2, 16). The observed association between IR surrogates and aortic stiffness is consistent with pathophysiological hypotheses involving endothelial dysfunction, inflammation, oxidative stress, altered nitric oxide signaling, and vascular remodeling (24, 25). The TyG index combines fasting triglyceride and glucose measurements and is correlated with metabolic features of hepatic IR (8, 26). Its association with vascular outcomes has been reported across general and high-risk populations (13, 27). TyG-BMI additionally incorporates adiposity, whereas TG/HDL-C reflects a broader pattern of atherogenic dyslipidemia (10, 11, 28, 29). These differences are compatible with, but do not causally explain, the stronger association observed for TyG in the present cohort. However, this finding does not show that TyG is a more specific causal measure of IR. All three indices are composite markers that may reflect overlapping metabolic, inflammatory, and vascular pathways. None of the fully adjusted Q4-versus-Q1 comparisons was statistically significant. Categorizing continuous exposures can reduce information and statistical power (30, 31); therefore, the continuous per-SD analyses provide the primary basis for inference, whereas quartile analyses are supportive. Recent evidence from hypertensive populations also highlights the interrelated associations of dyslipidemia, BMI, fasting glucose, and smoking with cardiometabolic risk (12).

TyG alone showed only moderate discrimination for aortic stiffness. The candidate cutoff of 8.68 yielded an AUC of 0.752, sensitivity of 72.0%, specificity of 66.1%, PPV of 50.9%, and NPV of 82.9% in the modeling cohort. Performance was lower in the external validation cohort (AUC = 0.694; sensitivity = 63.6%; specificity = 63.2%; PPV = 45.7%; NPV = 78.2%). Thus, statistical significance should not be interpreted as sufficient clinical utility. The relatively low PPV corresponds to a substantial proportion of potential false-positive referrals, whereas the NPV may be more useful for preliminary triage. The threshold should be treated as exploratory and cannot replace cfPWV or guide treatment. Published thresholds are difficult to compare because populations, vascular measures, and outcome definitions differ (13, 32–36).

The model including TyG showed a modest increment in discrimination beyond the base model. Although the NRI and IDI were statistically significant, these measures are sensitive to model specification and do not demonstrate clinical benefit. XGBoost had the highest apparent AUC among the six machine-learning models, but its performance did not differ significantly from that of logistic regression. We therefore prioritized the more interpretable model. Machine-learning models do not consistently outperform conventional regression in clinical risk-stratification tasks (37). The present model should be considered a preliminary risk-stratification tool rather than a stand-alone diagnostic instrument. Comparisons with previous nomograms and web calculators (38, 39) provide context, but differences in populations, outcomes, model variables, and validation designs preclude direct performance ranking. A recent systematic review of cardiovascular AI and machine-learning applications similarly emphasized rigorous validation and clinical integration before routine deployment (40).

The association between TyG and aortic stiffness was directionally consistent across the prespecified subgroups. No statistically significant interactions were detected for age, sex, hypertension, diabetes, smoking, or medication-use strata. The modeling cohort included both women and men, and the TyG association was observed in both sex strata. However, the study was not powered to detect modest interactions. Meta-analytic evidence has also reported associations between TyG and arterial stiffness across different ages, sexes, and participant characteristics (41). Differences between cfPWV and peripheral measures such as baPWV, cAIx, PPA, and ABI should be considered when comparing studies. Further studies should use standardized cfPWV measurements rather than assume that results from one vascular index apply to another.

Sensitivity analyses showed that TyG and TyG-BMI remained associated with aortic stiffness after excluding participants with hypertension, diabetes, or both conditions. In contrast, the TG/HDL-C association was attenuated after hypertension was excluded. This pattern may reflect effect modification or residual confounding by blood pressure, medication exposure, or disease-related vascular remodeling; the cross-sectional design cannot distinguish these explanations. Previous studies in hypertensive and non-hypertensive populations reported broadly similar differences among IR surrogates (10, 42, 43). Internal cross-validation showed stable discrimination in the modeling cohort, but resampling does not eliminate optimism introduced by variable selection, model comparison, or cutoff derivation. These results are supportive rather than definitive.

TyG can be calculated from routine fasting lipid and glucose measurements without specialized equipment. It may therefore help prioritize individuals for confirmatory cfPWV assessment when cfPWV is not routinely available. The five-variable model may also support preliminary risk stratification. However, neither a TyG value of 8.68 nor the model should be used as a diagnostic criterion or to determine treatment. Clinical implementation requires prospective evidence that the approach improves referral decisions, patient outcomes, or net benefit.

Several limitations should be considered. First, the cross-sectional design precludes causal inference. It cannot establish whether medication exposure or other measured clinical variables preceded TyG elevation, or whether TyG elevation preceded aortic stiffening. Prospective studies with repeated measurements are needed. Second, both cohorts were recruited from health-management centers at two tertiary hospitals in Anhui Province, limiting geographic and institutional diversity. Detailed ethnic composition was not available for analysis, which limits assessment of transportability to populations with different ancestry backgrounds. Third, medication data were limited to current use of major categories. Drug class, dose, duration, adherence, treatment changes, lipid-lowering therapy such as statins, and some comorbid conditions were not comprehensively captured. Dietary patterns and physical activity were not systematically quantified, although polyphenol-rich diets and exercise behaviors have been associated with lipid and glucose metabolic profiles (44, 45). These incompletely measured or unmeasured factors leave potential for residual confounding. Fourth, variable selection and cutoff derivation were performed in the modeling cohort, which may have introduced optimism despite internal cross-validation. The external validation cohort was relatively small and was recruited from the same province during the same period. Finally, the five-variable equation underwent institutional external validation, but the graphical nomogram was not separately externally validated or recalibrated. Larger, geographically and ethnically diverse prospective cohorts with detailed medication, comorbidity, dietary, and activity data are needed before routine clinical implementation.

## 5 Conclusion

Among asymptomatic adults undergoing routine health examinations, the TyG index showed the strongest adjusted association with cfPWV-defined aortic stiffness among the three surrogate markers evaluated. A five-variable model including age, MAP, diabetes mellitus, smoking, and TyG showed good discrimination in the modeling cohort and an independent institutional validation cohort. However, the standalone TyG threshold showed only moderate discrimination, the incremental AUC improvement was modest, and the graphical nomogram was not separately externally validated. The findings do not establish temporal sequence, causality, or clinical benefit. Prospective validation and impact studies in larger, geographically and ethnically diverse populations are required before routine use.

## Data Availability

The raw data supporting the conclusions of this article will be made available by the authors, without undue reservation.

## Glossary

TyG: triglyceride-glucose
BMI: body mass index
TG: triglycerides
HDL-C: high-density lipoprotein cholesterol
IR: insulin resistance
TyG-BMI: triglyceride glucose-body mass index
TG/HDL-C: triglycerides to high-density lipoprotein cholesterol ratio
cfPWV: carotid-femoral pulse wave velocity
OR: odds ratio
SD: standard deviation
*Δ*AUC: change in area under the curve
NRI: net reclassification improvement
IDI: integrated discrimination improvement
MAP: mean arterial pressure
CVD: cardiovascular disease
ESC/ESH: European Society of Cardiology/European Society of Hypertension
NO: nitric oxide
SBP: systolic blood pressure
DBP: diastolic blood pressure
TC: total cholesterol
LDL-C: low-density lipoprotein cholesterol
ALT: alanine aminotransferase
AST: aspartate aminotransferase
eGFR: estimated glomerular filtration rate
UA: uric acid
RBC: red blood cell count
HGB: hemoglobin
PLT: platelet count
EPV: events per variable
LASSO: least absolute shrinkage and selection operator
CI: confidence interval
RCS: restricted cubic spline
ROC: receiver operating characteristic
AUC: area under the receiver operating characteristic curve
IQR: interquartile range
AS: aortic stiffness
FBG: fasting blood glucose
SVM: support vector machine
VIF: variance inflation factor
PPV: positive predictive value
NPV: negative predictive value
PKC: protein kinase C
RAAS: renin-angiotensin-aldosterone system
ML: machine learning
XGBoost: extreme gradient boosting
LR: logistic regression
baPWV: brachial-ankle pulse wave velocity
CAVI: cardio-ankle vascular index
cAIx: central augmentation index
PPA: pulse pressure amplification
ABI: ankle-brachial index.
