# Supplementary material for: Association and discriminative value of insulin resistance surrogate markers for aortic stiffness in asymptomatic adults
Source: Front Cardiovasc Med. 2026 Sep 28;13:1920101. doi: 10.3389/fcvm.2026.1920101 (PMC13654755; doi:10.3389/fcvm.2026.1920101)
Supplement: Supplementary file 1 [file Datasheet1.docx]

**Supplementary Material**

As shown in Supplementary Table S1, no significant differences were observed in any baseline characteristics between the modeling cohort (n=457) and the validation cohort (n=101), including age (P=0.843), gender (P=0.363), BMI (P=0.544), blood pressure indices (SBP: P=0.948; DBP: P=0.729; MAP: P=0.783), lipid profiles, glucose levels, liver and kidney function markers, and the prevalence of aortic stiffness (P=0.977). These results indicate that the two cohorts were well balanced, supporting the validity of external validation.

**Table S1. Comparison of Baseline Characteristics Between the Modeling and Validation Cohorts**

| **Characteristic** | **Modeling Cohort (n=457)** | **Validation Cohort (n=101)** | **P value** |
| --- | --- | --- | --- |
| Age (years) | 53.0 [36.0, 61.0] | 53.0 [33.0, 62.0] | 0.843 |
| Gender (Male), n (%) | 226 (49.5%) | 55 (54.5%) | 0.363 |
| BMI (kg/m²) | 23.7 ± 3.6 | 23.5 ± 3.7 | 0.544 |
| SBP (mmHg) | 124.0 [114.0, 134.0] | 124.0 [116.0, 131.0] | 0.948 |
| DBP (mmHg) | 75.0 [69.0, 83.0] | 77.0 [69.0, 82.0] | 0.729 |
| MAP (mmHg) | 92.0 [84.7, 99.0] | 92.0 [84.0, 98.3] | 0.783 |
| Hypertension, n (%) | 141 (30.9%) | 34 (33.7%) | 0.582 |
| Diabetes, n (%) | 36 (7.9%) | 6 (5.9%) | 0.504 |
| Smoking, n (%) | 65 (14.2%) | 19 (18.8%) | 0.243 |
| Drinking, n (%) | 69 (15.7%) | 19 (19.4%) | 0.375 |
| TG (mg/dL) | 111.6 [79.7, 161.2] | 111.6 [83.3, 150.6] | 0.912 |
| TC (mg/dL) | 193.8 ± 40.2 | 187.5 ± 39.7 | 0.155 |
| HDL-C (mg/dL) | 54.5 [47.4, 62.4] | 51.8 [46.8, 59.9] | 0.093 |
| LDL-C (mg/dL) | 128.6 ± 30.4 | 124.0 ± 30.3 | 0.174 |
| FBG (mg/dL) | 94.4 [88.3, 106.7] | 96.2 [89.6, 104.2] | 0.506 |
| ALT (U/L) | 19.5 [14.0, 27.7] | 20.0 [15.4, 31.4] | 0.164 |
| AST (U/L) | 22.0 [19.0, 26.0] | 23.0 [20.0, 26.8] | 0.179 |
| eGFR (mL/min/1.73m²) | 105.0 ± 16.6 | 106.9 ± 17.1 | 0.292 |
| Creatinine (μmol/L) | 66.9 [58.0, 78.2] | 66.4 [56.9, 77.4] | 0.805 |
| Uric acid (μmol/L) | 344.1 ± 93.4 | 348.2 ± 80.0 | 0.684 |
| RBC (×10¹²/L) | 4.7 ± 0.5 | 4.7 ± 0.5 | 0.212 |
| Hemoglobin (g/L) | 140.0 [130.0, 151.0] | 144.0 [131.0, 153.0] | 0.409 |
| Platelet (×10⁹/L) | 223.0 ± 59.8 | 225.2 ± 56.6 | 0.741 |
| TyG Index | 8.6 [8.2, 9.0] | 8.6 [8.3, 8.9] | 0.967 |
| TyG-BMI | 206.6 ± 39.0 | 205.5 ± 41.0 | 0.786 |
| TG/HDL-C | 2.2 [1.4, 3.2] | 2.1 [1.4, 3.3] | 0.545 |
| cfPWV (m/s) | 9.3 [8.3, 10.5] | 9.2 [8.4, 10.5] | 0.689 |
| AS, n (%) | 150 (32.8%) | 33 (32.7%) | 0.977 |

*AS, aortic stiffness; BMI, body mass index; SBP, systolic blood pressure; DBP, diastolic blood pressure; MAP, mean arterial pressure; TG, triglycerides; TC, total cholesterol; HDL-C, high-density lipoprotein cholesterol; LDL-C, low-density lipoprotein cholesterol; FBG, fasting blood glucose; ALT, alanine aminotransferase; AST, aspartate aminotransferase; eGFR, estimated glomerular filtration rate; cfPWV, carotid-femoral pulse wave velocity. Data are presented as mean ± SD, median [IQR], or n (%). P values were derived from independent t-test, Mann-Whitney U test, or chi-square test as appropriate.*

As shown in Supplementary Table S2, significant differences were observed across TyG index quartiles for the majority of clinical and metabolic variables. Participants in higher TyG index quartiles were significantly older (Q1: 44.7 ± 16.7 years vs. Q4: 55.7 ± 14.0 years), more likely to be male (Q1: 39.1% vs. Q4: 62.3%), and had higher BMI, SBP, DBP, triglycerides, TC, LDL-C, FBG, ALT, AST, uric acid, RBC, and hemoglobin (all P<0.001). HDL-C and eGFR decreased progressively across quartiles (both P<0.001). The prevalence of smoking (P=0.012) and drinking (P=0.003) increased across quartiles, as did the prevalence of hypertension (Q1: 13.9% vs. Q4: 46.5%, P<0.001) and diabetes (Q1: 0.0% vs. Q4: 24.6%, P<0.001). Creatinine (P=0.126) and platelet count (P=0.124) did not differ significantly across quartiles. Carotid-femoral pulse wave velocity (cfPWV) increased from 8.5 [7.7, 9.2] m/s in Q1 to 10.0 [9.2, 11.6] m/s in Q4 (P<0.001), and the prevalence of aortic stiffness (AS) rose markedly from 13.0% in Q1 to 52.6% in Q4 (P<0.001).

**Table S2. Baseline Characteristics of Participants According to Quartiles of TyG Index**

| **Characteristic** | **Total (n=457)** | **Q1 (n=115)** | **Q2 (n=114)** | **Q3 (n=114)** | **Q4 (n=114)** | **P value** |
| --- | --- | --- | --- | --- | --- | --- |
| **TyG Index** | 8.6 [8.2, 9.0] | 8.0 [7.9, 8.2] | 8.4 [8.3, 8.5] | 8.8 [8.7, 8.9] | 9.3 [9.2, 9.6] | <0.001 |
| Age (years) | 50.7 ± 16.3 | 44.7 ± 16.7 | 50.0 ± 16.4 | 52.3 ± 16.0 | 55.7 ± 14.0 | <0.001 |
| Gender (Male), n (%) | 226 (49.5%) | 45 (39.1%) | 51 (44.7%) | 59 (51.8%) | 71 (62.3%) | 0.003 |
| BMI (kg/m²) | 23.7 ± 3.6 | 22.0 ± 3.0 | 22.7 ± 3.0 | 24.9 ± 4.0 | 25.3 ± 3.0 | <0.001 |
| SBP (mmHg) | 124.9 ± 15.3 | 117.9 ± 13.4 | 122.9 ± 15.1 | 127.0 ± 13.9 | 131.7 ± 15.3 | <0.001 |
| DBP (mmHg) | 76.3 ± 10.0 | 71.7 ± 8.2 | 74.6 ± 9.5 | 78.0 ± 9.7 | 80.9 ± 10.1 | <0.001 |
| MAP (mmHg) | 92.5 ± 10.9 | 87.1 ± 9.2 | 90.7 ± 10.4 | 94.3 ± 10.2 | 97.8 ± 11.0 | <0.001 |
| Hypertension, n (%) | 141 (30.9%) | 16 (13.9%) | 30 (26.3%) | 42 (36.8%) | 53 (46.5%) | <0.001 |
| Diabetes, n (%) | 36 (7.9%) | 0 (0.0%) | 4 (3.5%) | 4 (3.5%) | 28 (24.6%) | <0.001 |
| Smoking, n (%) | 65 (14.2%) | 16 (13.9%) | 9 (7.9%) | 14 (12.3%) | 26 (22.8%) | 0.012 |
| Drinking, n (%) | 69 (15.7%) | 10 (8.8%) | 14 (13.0%) | 16 (14.5%) | 29 (26.9%) | 0.003 |
| TG (mg/dL) | 111.6 [79.7, 161.2] | 66.4 [58.9, 74.4] | 97.4 [85.9, 107.2] | 135.5 [125.1, 150.6] | 220.5 [178.0, 270.6] | <0.001 |
| TC (mg/dL) | 193.8 ± 40.2 | 177.5 ± 35.5 | 188.7 ± 38.1 | 203.4 ± 40.2 | 205.7 ± 40.9 | <0.001 |
| HDL-C (mg/dL) | 54.5 [47.4, 62.4] | 59.2 [51.8, 68.6] | 55.3 [47.3, 62.6] | 52.4 [46.1, 60.7] | 50.7 [44.9, 57.6] | <0.001 |
| LDL-C (mg/dL) | 128.6 ± 30.4 | 114.1 ± 26.5 | 124.9 ± 28.1 | 138.3 ± 31.2 | 137.0 ± 29.5 | <0.001 |
| FBG (mg/dL) | 94.4 [88.3, 106.7] | 90.3 [85.6, 94.5] | 92.6 [87.2, 101.5] | 96.9 [89.8, 105.7] | 108.6 [95.4, 127.5] | <0.001 |
| ALT (U/L) | 19.5 [14.0, 27.7] | 16.0 [11.6, 20.9] | 17.0 [13.0, 22.8] | 21.0 [16.2, 30.2] | 26.6 [20.1, 39.0] | <0.001 |
| AST (U/L) | 22.0 [19.0, 26.0] | 20.6 [17.0, 23.9] | 22.0 [18.0, 24.3] | 22.1 [19.3, 26.2] | 25.0 [21.0, 30.0] | <0.001 |
| eGFR (mL/min/1.73m²) | 105.0 ± 16.6 | 109.9 ± 15.4 | 106.3 ± 16.5 | 102.6 ± 16.5 | 101.1 ± 16.7 | <0.001 |
| Creatinine (μmol/L) | 66.9 [58.0, 78.2] | 63.6 [57.7, 77.3] | 66.2 [55.7, 76.2] | 67.1 [59.4, 79.2] | 69.5 [58.8, 80.0] | 0.126 |
| Uric acid (μmol/L) | 344.1 ± 93.4 | 318.0 ± 85.4 | 321.5 ± 84.2 | 364.8 ± 100.4 | 372.3 ± 90.7 | <0.001 |
| RBC (×10¹²/L) | 4.7 ± 0.5 | 4.5 ± 0.5 | 4.6 ± 0.5 | 4.7 ± 0.4 | 4.8 ± 0.4 | <0.001 |
| Hemoglobin (g/L) | 140.4 ± 14.8 | 136.0 ± 14.1 | 137.0 ± 15.5 | 143.1 ± 12.8 | 145.5 ± 14.6 | <0.001 |
| Platelet (×10⁹/L) | 219.0 [180.0, 257.0] | 210.0 [176.0, 248.5] | 221.5 [191.2, 258.2] | 231.5 [184.0, 274.0] | 213.0 [174.0, 248.0] | 0.124 |
| cfPWV (m/s) | 9.2 [8.3, 10.5] | 8.5 [7.7, 9.2] | 9.1 [8.0, 9.8] | 9.4 [8.7, 10.8] | 10.0 [9.2, 11.6] | <0.001 |
| AS, n (%) | 145 (31.7%) | 15 (13.0%) | 25 (21.9%) | 45 (39.5%) | 60 (52.6%) | <0.001 |

*Note: Abbreviations are the same as in Supplementary Table S1.*

Table S3 presents baseline characteristics stratified by TyG-BMI quartiles. Higher TyG-BMI quartiles were significantly associated with older age (P<0.001), male sex (Q1: 33.9% vs. Q4: 65.8%, P<0.001), and higher SBP, DBP, triglycerides, TC, LDL-C, FBG, ALT, AST, creatinine, uric acid, RBC, and hemoglobin (all P<0.001). eGFR decreased significantly with higher TyG-BMI quartiles (P<0.001). HDL-C was inversely associated with TyG-BMI quartiles (P<0.001). The prevalence of smoking (P=0.017) and drinking (P=0.001) increased across quartiles. The prevalence of hypertension (Q1: 14.8% vs. Q4: 43.9%, P<0.001) and diabetes (Q1: 1.7% vs. Q4: 15.8%, P<0.001) increased progressively across quartiles. Platelet count did not differ significantly across quartiles (P=0.732). cfPWV increased from 8.3 [7.7, 9.5] m/s in Q1 to 9.6 [9.0, 10.9] m/s in Q4 (P<0.001), and the prevalence of AS rose from 18.3% in Q1 to 44.7% in Q4 (P<0.001).

**Table S3. Baseline Characteristics of Participants According to Quartiles of TyG-BMI**

| **Characteristic** | **Total (n=457)** | **Q1 (n=115)** | **Q2 (n=114)** | **Q3 (n=114)** | **Q4 (n=114)** | **P value** |
| --- | --- | --- | --- | --- | --- | --- |
| **TyG-BMI** | 201.8 [177.9, 229.4] | 163.8 [154.9, 170.6] | 192.7 [186.7, 197.2] | 215.8 [209.2, 222.3] | 252.4 [237.7, 268.3] | <0.001 |
| Age (years) | 50.7 ± 16.3 | 45.2 ± 17.8 | 51.3 ± 14.9 | 53.9 ± 15.3 | 52.3 ± 15.7 | <0.001 |
| Gender (Male), n (%) | 226 (49.5%) | 39 (33.9%) | 52 (45.6%) | 60 (52.6%) | 75 (65.8%) | <0.001 |
| SBP (mmHg) | 124.9 ± 15.3 | 117.3 ± 15.1 | 123.5 ± 14.0 | 127.5 ± 13.7 | 131.2 ± 14.8 | <0.001 |
| DBP (mmHg) | 76.3 ± 10.0 | 71.3 ± 9.3 | 75.2 ± 9.1 | 77.8 ± 8.7 | 80.9 ± 10.4 | <0.001 |
| MAP (mmHg) | 92.5 ± 10.9 | 86.7 ± 10.5 | 91.3 ± 9.7 | 94.4 ± 9.3 | 97.7 ± 11.1 | <0.001 |
| Hypertension, n (%) | 141 (30.9%) | 17 (14.8%) | 31 (27.2%) | 43 (37.7%) | 50 (43.9%) | <0.001 |
| Diabetes, n (%) | 36 (7.9%) | 2 (1.7%) | 8 (7.0%) | 8 (7.0%) | 18 (15.8%) | 0.001 |
| Smoking, n (%) | 65 (14.2%) | 15 (13.0%) | 10 (8.8%) | 14 (12.3%) | 26 (22.8%) | 0.017 |
| Drinking, n (%) | 69 (15.7%) | 6 (5.5%) | 15 (13.6%) | 22 (20.0%) | 26 (23.9%) | 0.001 |
| TG (mg/dL) | 111.6 [79.7, 161.2] | 72.6 [61.1, 89.9] | 98.8 [79.9, 118.7] | 135.5 [102.1, 167.2] | 181.1 [132.6, 261.9] | <0.001 |
| TC (mg/dL) | 193.8 ± 40.2 | 184.3 ± 42.1 | 189.7 ± 36.3 | 201.7 ± 36.9 | 199.7 ± 43.1 | 0.002 |
| HDL-C (mg/dL) | 54.5 [47.4, 62.4] | 60.3 [52.1, 70.2] | 56.1 [48.4, 64.1] | 53.6 [47.2, 59.8] | 48.7 [41.8, 55.3] | <0.001 |
| LDL-C (mg/dL) | 128.6 ± 30.4 | 118.3 ± 30.6 | 125.3 ± 28.1 | 136.3 ± 28.8 | 134.6 ± 30.9 | <0.001 |
| FBG (mg/dL) | 94.4 [88.3, 106.7] | 91.2 [85.9, 96.8] | 93.2 [88.7, 102.0] | 96.9 [89.6, 108.3] | 102.3 [92.5, 117.8] | <0.001 |
| ALT (U/L) | 19.5 [14.0, 27.7] | 15.1 [11.0, 20.6] | 18.0 [13.0, 23.0] | 21.0 [15.5, 30.3] | 26.9 [20.7, 39.0] | <0.001 |
| AST (U/L) | 22.0 [19.0, 26.0] | 20.0 [17.0, 23.4] | 21.5 [19.0, 24.3] | 22.3 [19.0, 26.8] | 24.4 [21.7, 31.8] | <0.001 |
| eGFR (mL/min/1.73m²) | 105.0 ± 16.6 | 110.4 ± 17.1 | 105.5 ± 15.6 | 101.6 ± 15.4 | 102.4 ± 16.9 | <0.001 |
| Creatinine (μmol/L) | 66.9 [58.0, 78.2] | 62.0 [56.8, 74.2] | 65.8 [55.8, 76.3] | 66.8 [59.5, 79.8] | 72.4 [61.3, 82.7] | <0.001 |
| Uric acid (μmol/L) | 344.1 ± 93.4 | 299.0 ± 72.9 | 336.7 ± 81.6 | 358.6 ± 97.8 | 382.4 ± 99.1 | <0.001 |
| RBC (×10¹²/L) | 4.7 ± 0.5 | 4.5 ± 0.5 | 4.6 ± 0.4 | 4.7 ± 0.4 | 4.8 ± 0.5 | <0.001 |
| Hemoglobin (g/L) | 140.4 ± 14.8 | 133.5 ± 14.4 | 138.5 ± 13.9 | 143.0 ± 12.9 | 146.6 ± 14.8 | <0.001 |
| Platelet (×10⁹/L) | 219.0 [180.0, 257.0] | 217.0 [181.0, 247.4] | 227.5 [189.2, 258.8] | 214.0 [174.0, 260.2] | 220.5 [178.0, 256.8] | 0.732 |
| cfPWV (m/s) | 9.2 [8.3, 10.5] | 8.3 [7.7, 9.5] | 9.0 [8.3, 10.1] | 9.5 [8.7, 11.0] | 9.6 [9.0, 10.9] | <0.001 |
| AS, n (%) | 145 (31.7%) | 21 (18.3%) | 30 (26.3%) | 43 (37.7%) | 51 (44.7%) | <0.001 |

*Note: Abbreviations are the same as in Table S1.*

Table S4 shows baseline characteristics across TG/HDL-C quartiles. In contrast to TyG index and TyG-BMI, age did not differ significantly across TG/HDL-C quartiles (P=0.107), suggesting that this ratio captures lipid-centric metabolic risk more specifically. Male sex (Q1: 33.9% vs. Q4: 68.4%), BMI, SBP, DBP, triglycerides, LDL-C, FBG, ALT, AST, creatinine, uric acid, RBC, hemoglobin, and platelet count (all P<0.05) differed significantly across quartiles. HDL-C decreased progressively from Q1 to Q4 (P<0.001). TC (P=0.085) and eGFR (P=0.072) did not differ significantly across TG/HDL-C quartiles. The prevalence of hypertension (P=0.013) and diabetes (Q1: 1.7% vs. Q4: 18.4%, P<0.001) increased significantly across quartiles. Smoking (P=0.009) and drinking (P=0.021) also differed significantly across quartiles. cfPWV increased progressively from 8.8 [7.7, 9.8] m/s in Q1 to 9.7 [9.0, 11.1] m/s in Q4 (P<0.001), and the prevalence of AS rose from 21.7% in Q1 to 46.5% in Q4 (P<0.001).

**Table S4. Baseline Characteristics of Participants According to Quartiles of TG/HDL-C**

| **Characteristic** | **Total (n=457)** | **Q1 (n=115)** | **Q2 (n=114)** | **Q3 (n=114)** | **Q4 (n=114)** | **P value** |
| --- | --- | --- | --- | --- | --- | --- |
| **TG/HDL-C** | 2.2 [1.4, 3.2] | 1.0 [0.9, 1.2] | 1.7 [1.5, 1.9] | 2.5 [2.3, 2.8] | 4.4 [3.7, 5.7] | <0.001 |
| Age (years) | 50.7 ± 16.3 | 47.5 ± 17.2 | 52.2 ± 16.8 | 50.9 ± 16.3 | 52.1 ± 14.4 | 0.107 |
| Gender (Male), n (%) | 226 (49.5%) | 39 (33.9%) | 48 (42.1%) | 61 (53.5%) | 78 (68.4%) | <0.001 |
| BMI (kg/m²) | 23.7 ± 3.6 | 21.7 ± 2.5 | 23.0 ± 4.1 | 24.7 ± 3.2 | 25.5 ± 2.9 | <0.001 |
| SBP (mmHg) | 124.9 ± 15.3 | 120.1 ± 15.0 | 124.2 ± 13.3 | 125.3 ± 16.3 | 129.9 ± 14.8 | <0.001 |
| DBP (mmHg) | 76.3 ± 10.0 | 72.1 ± 9.0 | 75.9 ± 8.7 | 76.7 ± 10.1 | 80.5 ± 10.4 | <0.001 |
| MAP (mmHg) | 92.5 ± 10.9 | 88.1 ± 10.2 | 92.0 ± 9.1 | 92.9 ± 11.4 | 97.0 ± 11.1 | <0.001 |
| Hypertension, n (%) | 141 (30.9%) | 27 (23.5%) | 28 (24.6%) | 40 (35.1%) | 46 (40.4%) | 0.013 |
| Diabetes, n (%) | 36 (7.9%) | 2 (1.7%) | 7 (6.1%) | 6 (5.3%) | 21 (18.4%) | <0.001 |
| Smoking, n (%) | 65 (14.2%) | 13 (11.3%) | 9 (7.9%) | 17 (14.9%) | 26 (22.8%) | 0.009 |
| Drinking, n (%) | 69 (15.7%) | 12 (10.5%) | 13 (12.1%) | 17 (15.3%) | 27 (25.2%) | 0.021 |
| TG (mg/dL) | 111.6 [79.7, 161.2] | 65.5 [58.5, 75.7] | 94.8 [85.0, 108.7] | 133.7 [118.7, 148.8] | 217.0 [174.7, 270.6] | <0.001 |
| TC (mg/dL) | 193.8 ± 40.2 | 186.4 ± 40.7 | 193.0 ± 39.1 | 199.3 ± 42.8 | 196.7 ± 37.5 | 0.085 |
| HDL-C (mg/dL) | 54.5 [47.4, 62.4] | 64.2 [57.6, 73.9] | 55.7 [49.6, 61.8] | 51.0 [46.5, 57.0] | 46.4 [41.0, 53.9] | <0.001 |
| LDL-C (mg/dL) | 128.6 ± 30.4 | 117.6 ± 30.3 | 129.1 ± 29.7 | 135.6 ± 32.4 | 132.1 ± 26.3 | <0.001 |
| FBG (mg/dL) | 94.4 [88.3, 106.7] | 92.3 [86.3, 100.6] | 94.1 [87.4, 104.1] | 95.1 [89.0, 105.3] | 100.5 [91.8, 120.1] | <0.001 |
| ALT (U/L) | 19.5 [14.0, 27.7] | 15.0 [11.4, 19.1] | 18.0 [13.1, 24.1] | 22.3 [16.1, 31.0] | 26.0 [19.0, 39.0] | <0.001 |
| AST (U/L) | 22.0 [19.0, 26.0] | 20.0 [17.0, 23.0] | 22.0 [18.8, 25.0] | 23.0 [20.0, 26.0] | 24.0 [20.0, 29.8] | <0.001 |
| eGFR (mL/min/1.73m²) | 105.0 ± 16.6 | 108.1 ± 16.5 | 103.7 ± 16.3 | 105.4 ± 15.9 | 102.8 ± 17.3 | 0.072 |
| Creatinine (μmol/L) | 66.9 [58.0, 78.2] | 61.6 [56.5, 75.2] | 66.6 [57.3, 77.8] | 65.9 [58.9, 77.1] | 73.8 [61.5, 81.5] | 0.002 |
| Uric acid (μmol/L) | 344.1 ± 93.4 | 304.1 ± 79.1 | 326.8 ± 83.6 | 358.6 ± 95.5 | 387.1 ± 93.8 | <0.001 |
| RBC (×10¹²/L) | 4.7 ± 0.5 | 4.4 ± 0.5 | 4.6 ± 0.5 | 4.7 ± 0.5 | 4.8 ± 0.4 | <0.001 |
| Hemoglobin (g/L) | 140.4 ± 14.8 | 134.5 ± 13.3 | 138.6 ± 14.5 | 141.9 ± 14.7 | 146.6 ± 14.2 | <0.001 |
| Platelet (×10⁹/L) | 219.0 [180.0, 257.0] | 207.0 [176.0, 248.5] | 223.5 [183.2, 250.8] | 232.5 [191.8, 273.5] | 214.0 [172.5, 253.8] | 0.042 |
| cfPWV (m/s) | 9.2 [8.3, 10.5] | 8.8 [7.7, 9.8] | 9.1 [8.3, 10.3] | 9.3 [8.6, 10.7] | 9.7 [9.0, 11.1] | <0.001 |
| AS, n (%) | 145 (31.7%) | 25 (21.7%) | 34 (29.8%) | 33 (28.9%) | 53 (46.5%) | <0.001 |

*Note: Abbreviations are the same as in Table S1.*

In the modeling cohort, grid search with 10-fold cross-validation was employed to identify the optimal hyperparameters for each of the six models. The final hyperparameter configurations are summarized in Supplementary Table S5.

## **Table S5. Optimal Hyperparameters of Six ML Models in Aortic Stiffness Risk Prediction.**

| Model | Optimal parameters |
| --- | --- |
| Logistic Regression | 'C': 10.0, 'class_weight': None, 'penalty': 'l1', 'solver': 'liblinear' |
| XGBoost | 'colsample_bytree': 1.0, 'gamma': 0.5, 'learning_rate': 0.2, 'max_depth': 5, 'min_child_weight': 1, 'n_estimators': 300, 'reg_alpha': 0.1, 'reg_lambda': 10, 'scale_pos_weight': 1, 'subsample': 0.9 |
| Decision Tree | 'ccp_alpha': 0.005, 'class_weight': None, 'criterion': 'entropy', 'max_depth': 5, 'min_samples_leaf': 5, 'min_samples_split': 50 |
| Random Forest | 'bootstrap': True, 'ccp_alpha': 0.005, 'class_weight': 'balanced', 'max_depth': 5, 'max_features': None, 'min_samples_leaf': 1, 'min_samples_split': 10, 'n_estimators': 300 |
| SVM | 'C': 50.0, 'class_weight': 'balanced', 'gamma': 0.05, 'kernel': 'rbf' |
| Naive Bayes | 'var_smoothing': 1e-06 |

*Note: Values are point estimates from 10-fold cross-validation. AUC: area under the receiver operating characteristic curve; PPV: positive predictive value; NPV: negative predictive value.*

To evaluate the robustness of the primary findings, sensitivity analyses were performed under four alternative analytical scenarios: (1) excluding participants with hypertension (n=299, AS events=46); (2) excluding participants with diabetes mellitus (n=406, AS events=114); and (3) simultaneously excluding participants with both hypertension and diabetes mellitus (n=284, AS events=36). All odds ratios are expressed per standard deviation increment. As shown in Supplementary Table S6, TyG index remained significantly and positively associated with aortic stiffness across all four scenarios (OR range: 2.65–2.88; all P≤0.008). TyG-BMI demonstrated consistent statistical significance in scenarios (1), (2), and (3) (all P<0.05). For TG/HDL-C, significant associations were observed in the complete-case analysis (OR=1.28, 95% CI: 1.05–1.56, P=0.032) and after excluding diabetes (OR=1.53, 95% CI: 1.01–2.31, P=0.044); the association did not reach statistical significance after excluding hypertension (P=0.097) or both conditions (P=0.139).

## **Table S6. Sensitivity Analyses of Insulin Resistance Indices and Aortic Stiffness**

| Analysis | TyG Index OR (95% CI) | P value | TyG-BMI OR (95% CI) | P value | TG/HDL-C OR (95% CI) | P value |
| --- | --- | --- | --- | --- | --- | --- |
| Complete case | 3.91 (1.87-8.15) | < 0.001 | 1.01 (1.00-1.03) | 0.014 | 1.28 (1.05-1.56) | 0.014 |
| Excluding hypertension | 2.65 (1.38–5.07) | 0.003 | 1.88 (1.09–3.25) | 0.024 | 1.79 (0.90–3.56) | 0.097 |
| Excluding diabetes | 2.12 (1.30–3.46) | 0.003 | 1.67 (1.06–2.63) | 0.026 | 1.53 (1.01–2.31) | 0.044 |
| Excluding HTN and diabetes | 2.88 (1.31–6.32) | 0.008 | 2.05 (1.13–3.70) | 0.018 | 1.84 (0.82–4.15) | 0.139 |

*Note: ORs for insulin resistance indices are expressed per standard deviation (SD) increment. Models are adjusted for Age, Gender, Smoking, Alcohol, History of Hypertension, SBP, DBP, TC, LDL-C, ALT, AST, eGFR, Creatinine, UA, RBC, HGB, and PLT. HTN, hypertension.*

Ten-fold stratified cross-validation was performed on the modeling cohort, yielding a mean AUC of 0.948, mean accuracy of 0.886, mean sensitivity of 0.829, mean specificity of 0.914, mean PPV of 0.833, mean NPV of 0.920, and mean Brier score of 0.085. The low standard deviations across folds indicate stable and reproducible model performance, supporting the robustness of the five-variable prediction model (age, MAP, diabetes mellitus, smoking, and TyG index) across different data partitions. These cross-validation results are consistent with the external validation AUC of 0.909 reported in the primary analysis, further confirming the generalizability of the model (**Table S7, Figure S1**)

## **Table S7. Per-Fold Performance Metrics of the Logistic Regression Model in 10-Fold Stratified Cross-Validation.**

| **Fold** | **AUC** | **Accuracy** | **Sensitivity** | **Specificity** | **PPV** | **NPV** | **Brier Score** |
| --- | --- | --- | --- | --- | --- | --- | --- |
| 1 | 0.864 | 0.804 | 0.786 | 0.812 | 0.647 | 0.897 | 0.151 |
| 2 | 0.987 | 0.957 | 0.857 | 1.000 | 1.000 | 0.941 | 0.051 |
| 3 | 0.929 | 0.848 | 0.867 | 0.839 | 0.722 | 0.929 | 0.103 |
| 4 | 0.935 | 0.913 | 0.800 | 0.968 | 0.923 | 0.909 | 0.092 |
| 5 | 0.953 | 0.891 | 0.733 | 0.968 | 0.917 | 0.882 | 0.074 |
| 6 | 0.974 | 0.891 | 0.733 | 0.968 | 0.917 | 0.882 | 0.070 |
| 7 | 0.972 | 0.935 | 0.867 | 0.968 | 0.929 | 0.938 | 0.066 |
| 8 | 0.977 | 0.911 | 0.929 | 0.903 | 0.812 | 0.966 | 0.063 |
| 9 | 0.917 | 0.822 | 0.857 | 0.806 | 0.667 | 0.926 | 0.113 |
| 10 | 0.968 | 0.889 | 0.857 | 0.903 | 0.800 | 0.933 | 0.066 |
| **Mean** | **0.948** | **0.886** | **0.829** | **0.914** | **0.833** | **0.920** | **0.085** |
| **SD** | 0.037 | 0.048 | 0.063 | 0.072 | 0.123 | 0.027 | 0.030 |

*Figure S1. Receiver operating characteristic (ROC) curves for each of the 10 cross-validation folds (colored lines) and the mean ROC curve (dashed black line) with ±1 standard deviation band (grey shading). Mean AUC = 0.948 ± 0.037.*
